# Using textons to rank crystallization droplets by the likely presence of crystals

**DOI:** 10.1107/S1399004714017581

**Published:** 2014-09-27

**Authors:** Jia Tsing Ng, Carien Dekker, Markus Kroemer, Michael Osborne, Frank von Delft

**Affiliations:** a Structural Genomics Consortium, University of Oxford, Roosevelt Drive, Oxford OX3 7DQ, England; b Novartis Institute for Biomedical Research, Novartis Campus, Postfach, CH-4056 Basel, Switzerland; c Department of Engineering Science, University of Oxford, Parks Road, Oxford OX1 3PJ, England; d Diamond Light Source Ltd, Harwell Science and Innovation Campus, Didcot OX11 0QX, England; e Department of Biochemistry, University of Johannesburg, Auckland Park 2006, South Africa

**Keywords:** textons, crystallization, identification of crystals

## Abstract

The visual inspection of crystallization experiments is an important yet time-consuming and subjective step in X-ray crystallo­graphy. Previously published studies have focused on automatically classifying crystallization droplets into distinct but ultimately arbitrary experiment outcomes; here, a method is described that instead ranks droplets by their likelihood of containing crystals or microcrystals, thereby prioritizing for visual inspection those images that are most likely to contain useful information. The use of textons is introduced to describe crystallization droplets objectively, allowing them to be scored with the posterior probability of a random forest classifier trained against droplets manually annotated for the presence or absence of crystals or microcrystals. Unlike multi-class classification, this two-class system lends itself naturally to unidirectional ranking, which is most useful for assisting sequential viewing because images can be arranged simply by using these scores: this places droplets with probable crystalline behaviour early in the viewing order. Using this approach, the top ten wells included at least one human-annotated crystal or microcrystal for 94% of the plates in a data set of 196 plates imaged with a Minstrel HT system. The algorithm is robustly transferable to at least one other imaging system: when the parameters trained from Minstrel HT images are applied to a data set imaged by the Rock Imager system, human-annotated crystals ranked in the top ten wells for 90% of the plates. Because rearranging images is fundamental to the approach, a custom viewer was written to seamlessly support such ranked viewing, along with another important output of the algorithm, namely the shape of the curve of scores, which is itself a useful overview of the behaviour of the plate; additional features with known usefulness were adopted from existing viewers. Evidence is presented that such ranked viewing of images allows faster but more accurate evaluation of drops, in particular for the identification of microcrystals.

## Abstract 2

A robust and transferable algorithm is presented to objectively describe and rank robotically captured images of crystallization droplets according to their likelihood of crystalline behaviour for the efficient and accurate identification of successful crystallization.

## 1. Introduction   

The development and widespread deployment of robotics for high-throughput crystallization in the last decades has led to experimenters typically being confronted with evaluating very large numbers of crystallization trials, which is challenging even though the images are electronically captured (McPherson & Gavira, 2014 ▶). While such a high throughput facilitates the efficient and automated sampling of chemical space for crystallizing conditions, by itself it does not answer the question of whether the protein crystallizes; this requires crystals to be both present and reliably identified, although experienced practitioners can apparently also infer this from precipitation behaviours, but presumably less reliably so (Newman *et al.*, 2012 ▶). Thus, if a crystallization experiment is considered to be an assay of the suitability of a protein preparation for structural studies, a quick yet robust assessment of the presence of crystallinity can be hugely beneficial in identifying the gene construct or protein preparation that is most likely to crystallize. Robotic imaging of droplets offers an opportunity to do this, and we present an algorithm that seeks to exploit it.

A significant body of research exists on accurately identifying crystals from robotically captured images. Early work used edge detection solely to detect crystals (Ward *et al.*, 1988 ▶; Zuk & Ward, 1991 ▶), which was later extended to the auto-classification of experimental outcomes into various numbers of classes, for example ‘clear’, ‘precipitate’ or ‘crystalline behaviour’, with information derived from a diverse range of texture-analysis methods and/or edge detection, using off-the-shelf machine-learning algorithms. While Wilson (2002 ▶) and Bern *et al.* (2004 ▶) used only edge-based features, most work focused on texture analysis, either on its own or in combination with edge-based features, including grey-level occurrence matrices (GLCMs; Spraggon *et al.*, 2002 ▶; Zhu *et al.*, 2004 ▶; Cumbaa & Jurisica, 2010 ▶) and spectral methods such as Fourier transform (Walker *et al.*, 2007 ▶) and wavelet modelling (Buchala & Wilson, 2008 ▶; Liu *et al.*, 2008 ▶; Watts *et al.*, 2008 ▶). More recently, Lekamge *et al.* (2013 ▶) used time-series information by calculating GLCMs from the difference images of consecutive inspections. There was also a general trend that started with deriving features globally from the whole droplet (Spraggon *et al.*, 2002 ▶; Wilson, 2002 ▶), which shifted to identifying local features from subsections of an image (Bern *et al.*, 2004 ▶; Zhu *et al.*, 2004 ▶; Kawabata *et al.*, 2006 ▶; Pan *et al.*, 2006 ▶; Liu *et al.*, 2008 ▶), but later returned again to global features (Walker *et al.*, 2007 ▶; Buchala & Wilson, 2008 ▶; Watts *et al.*, 2008 ▶; Cumbaa & Jurisica, 2010 ▶; Lekamge *et al.*, 2013 ▶). A variety of machine-learning techniques have been used, including the naïve Bayes classifier (Wilson, 2002 ▶), support vector machines (Kawabata *et al.*, 2006 ▶; Pan *et al.*, 2006 ▶; Buchala & Wilson, 2008 ▶) and neural networks (Spraggon *et al.*, 2002 ▶; Walker *et al.*, 2007 ▶; Buchala & Wilson, 2008 ▶; Watts *et al.*, 2008 ▶), but the majority of recent work has favoured decision tree-based methods (Bern *et al.*, 2004 ▶; Liu *et al.*, 2008 ▶; Cumbaa & Jurisica, 2010 ▶; Lekamge *et al.*, 2013 ▶). It is notable that in all of this body of work it has not been much explored how to present the output of the computations to experimenters most effectively.

In contrast to GLCM or spectral methods, textures can also be modelled by the joint distribution of filter response cluster centres, otherwise known as textons (Leung & Malik, 2001 ▶). Julesz (1981 ▶) first proposed that textons were linked to human perception by using second-order statistics of local primitive elements, which were later refined by Leung & Malik (2001 ▶) with Gaussian derivative filters for operation on grey-level images. The approach is based on the argument that textures, by definition, contain repeating elements which correlate similarly to certain defined image motifs; hence, they have been termed ‘response to image filters’. Thus, for a series of textures, the vectors of filter responses can be reduced to only a few prototypes, while the remaining are noisy variations of these prototypes. Textons have since been used in many application areas, including the identification of brain tumours (Islam *et al.*, 2013 ▶), the identication of skin regions for gesture detection (Medeiros *et al.*, 2013 ▶), the classification of malignant mass regions in mammograms (Li *et al.*, 2012 ▶), the classification of different real-world textures (Varma & Zisserman, 2005 ▶), the diagnosis of Alzheimer’s disease (Morgado *et al.*, 2013 ▶), the decription of iris images for ethnic classification (Qiu *et al.*, 2007 ▶) and counterfeit detection (Wei *et al.*, 2008 ▶), as well as three-dimensional face recognition (Zhong *et al.*, 2007 ▶). However, they have not yet been used for analysing crystallization experiments.

Identifying the droplet in the image (‘segmentation’) is important to avoid interpreting noise from regions outside the droplet. Approaches to the problem include edge detection (Spraggon *et al.*, 2002 ▶; Wilson, 2002 ▶; Bern *et al.*, 2004 ▶), deriving masks from wavelet transforms (Watts *et al.*, 2008 ▶), using plate-specific features (Pan *et al.*, 2006 ▶; Cumbaa & Jurisica, 2010 ▶) or simply using the centre or regions of an image (Kawabata *et al.*, 2006 ▶; Liu *et al.*, 2008 ▶). A more robust algorithm was developed by Vallotton *et al.* (2010 ▶), which transforms the image from Euclidean to polar coordinates centred at a point in the droplet, allowing a shortest-path algorithm to be used to trace the droplet boundary, resulting in a robust method with no constraints on the droplet shape, for example having to be close to a perfect circle.

Here, we revisit the problem of crystal recognition, both using recent techniques of texture analysis and drop identification and reconsidering how the results can be most usefully presented to allow quick identification of the propensity of a protein preparation to crystallize. Rather than classifying drops into discrete categories of experimental outcomes, which has consistently been insufficiently accurate in all reports, we circumvent the question by instead ranking drops based on their precipitation behaviour, thereby rearranging the viewing order in an experimentally meaningful way, as opposed to the common top-left to bottom-right viewing sequence of droplets that is merely an artefact of the image numbering. This is in the spirit of Liu *et al.* (2008 ▶), who sought to identify harvestable crystals greater than 10 µm, but our algorithm additionally identifies droplets with microcrystals and showers of crystals, which are equally interesting to the question of crystallizability. We show the enrichment of crystal-containing images early in the viewing order and how crystals and microcrystals are found more efficiently and accurately when viewed in rank order.

## 2. Methods   

**Figure 1:**
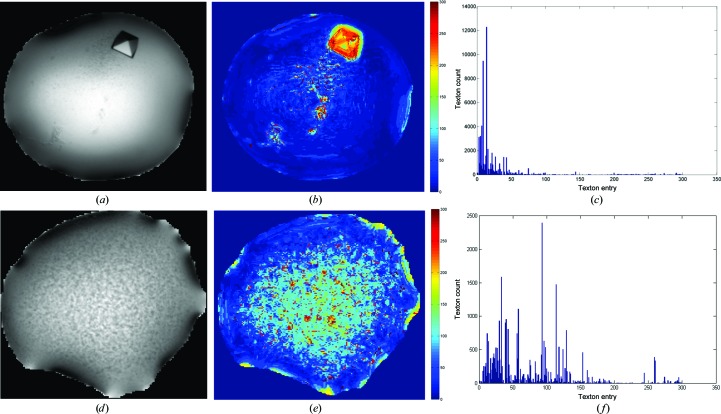
Counting patterns in a droplet. Examples of droplets with crystal (*a*) and crystalline precipitate (*d*). (*b*) and (*e*) show the artificial colouring of the respective droplets based on textons, where similar textures are coloured with the same colours; the associated frequency histograms of texton occurrences in the droplets are shown in (*c*) and (*f*).

Our approach entails first objectively characterizing crystallization droplets by describing and quantifying with textons the textural patterns that are are found in each droplet (Fig. 1 ▶), which yields a numerical descriptor in the form of a histogram of occurrence of different textons in the droplet. The frequency numbers are subsequently used as input to a random forest classifier, although not to classify the drops, which would entail selecting a classification threshold, but instead to obtain the posterior probability that is output by the classifier, which is used as a score to rank the droplets. The algorithms are described in detail in the following sections.

### 2.1. Texture-analysis method   

#### 2.1.1. Textons and filter banks   

**Figure 2:**
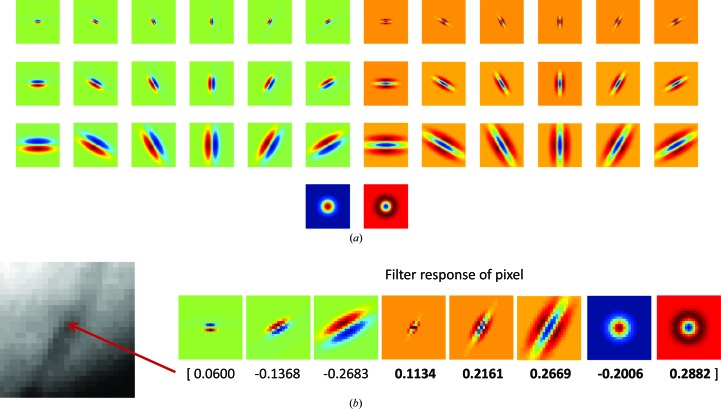
(*a*) The filter bank as proposed by Varma & Zisserman (2005 ▶) consists of edge (top three rows, left half) and bar (top three rows, right half) filters at six orientations and three scales, and two rotationally symmetric filters (Gaussian and Laplacian of Gaussian; bottom row). (*b*) Example of the filter response of a pixel, where only the maximum response of the edge and bar filters at each scale are kept, resulting a response vector with eight numbers.

Image filters are integral to the texton method, since textons are filter response cluster centres. Varma & Zisserman (2005 ▶) proposed a filter bank that is rotationally invariant, which contains edge filters and bar filters (at six orientations and three scales each), a Gaussian and a Laplacian of Gaussian filter (see Fig. 2 ▶ *a*). An image is filtered with all 38 filters in the filter bank, but only the maximum filter response for the edge and bar filters at each scale is recorded, resulting in a vector with eight (three edge, three bar, two Gaussians) rotation-invariant filter responses per pixel. An example of the final filter response vector is shown in Fig. 2 ▶(*b*). We chose this filter bank as it was shown by Varma and Zisserman to be the best filter bank when compared with the filter bank proposed by Leung & Malik (2001 ▶) and a ‘Gabor-like’ filter bank designed by Schmid (2001 ▶). This filter bank was used at half the scale originally proposed, which was found to be suitable for the precipitation patterns within our droplet images.

The texton method involves (i) building a texton dictionary containing unique texture prototypes and (ii) comparing the filter response of each pixel in a new image to this dictionary to find the closest texton and label the pixel with the corresponding texton label. The conventional way of building this dictionary is by taking a fixed number of textons from each class of textures and combining them to form the final dictionary. However, precipitation behaviour yields a continuum of patterns which are difficult to classify objectively by eye, and since no labelled data set is available in the community, we took a different approach to generate this dictionary, as outlined in the following section.

#### 2.1.2. Building the texton dictionary   

**Figure 3:**
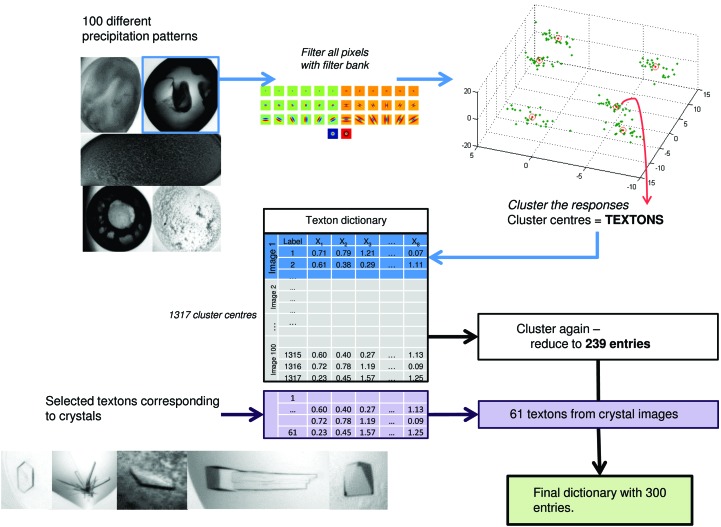
Process of generating the texton dictionary. 100 droplets with a wide range of patterns were selected; clusters of filter responses (textons) in these images were combined to form a dictionary of 1317 entries; to remove redundancy, the dictionary entries are clustered again and reduced to 239; selected textons corresponding to crystals were added to the dictionary to form the final dictionary with 300 textons.

To ensure that crystal-related textons are well represented, we used separate sets of 52 arbitrarily selected crystal-containing images and 100 precipitation images to cover a wide range of patterns (images are shown in Supplementary Figs. S1 and S2^1^). We found that these images were sufficient to produce the final dictionary and additional images did not give rise to new clusters. All images were scaled down by a factor of four for computational efficiency without compromising the final performance (data not shown). The final effective resolution of the images being filtered is around 4.5 µm per pixel length. Fig. 3 ▶ shows a diagram of this process.

For the set of 100 precipitation images, unlike most texton-generation schemes which use *k*-means clustering, for each image filter responses for all pixels were clustered using Gaussian mixture models with variational Bayes model selection (Corduneanu & Bishop, 2001 ▶), where no parameter selection was required, and the number of clusters arising from each image was determined by the data alone. Each image generally produced eight to 15 clusters. The resulting cluster centres (textons) of each image were concatenated and subsequently clustered again using Dirichlet process means (DP means; Kulis & Jordan, 2011 ▶) with λ = 0.5, where λ is the Euclidean distance threshold to start a new cluster, resulting in a dictionary with 239 entries. This cluster-and-cluster-again approach was required because the first clustering method was not configured to remove inter-image redundancy of patterns found in the selected images. Clusters were formed independently for each image; thus, similar patterns (for example clear regions) in different images will produce similar or overlapping cluster centres (textons). These redundant textons are pruned by the second clustering method, DP means, which clusters nearby points for a single representation but allows far-away single data points to form a new cluster with some penalty, rather than grouping them into the nearest cluster. Such properties of DP means are desirable because a far-away point in this case is not an outlier, but an actual texture seen in the training data set, and hence should be kept; this is in contrast to the first clustering stage, which sought to identify commonly occurring motifs, and hence single far-off data points corresponded to noise.

For the set of 52 crystal-containing images, filter responses for each image were similarly clustered with Gaussian mixture models with variational Bayes model selection. Crystal-related cluster centres for each image were selected and clustered by DP means with similar parameters as described previously. The resulting textons were added to the dictionary, producing the final texton dictionary with 300 entries. For the purpose of visual representation, the dictionary was arranged so that the first entry is the texton with the lowest magnitude, and all subsequent entries were sorted by their distance with respect to this texton. The dictionary is available with the software distribution.

#### 2.1.3. Generating features with the texton dictionary   

**Figure 4:**
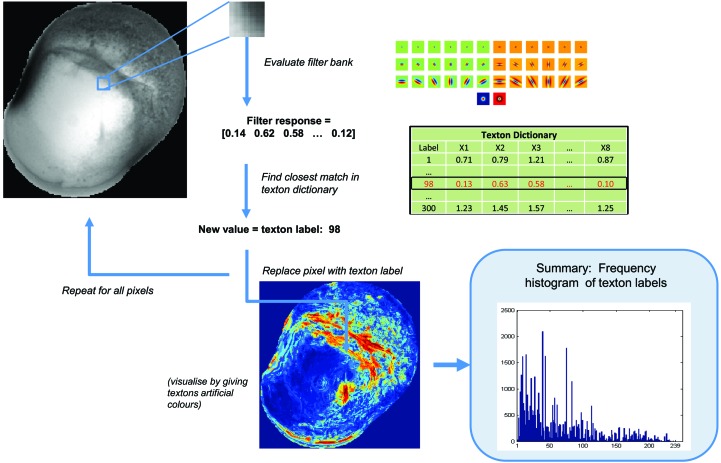
Calculating the texton distribution from an image. Given a new image, each pixel is filtered with the filter bank and its filter response is compared with the texton dictionary generated previously. The label of the closest match in the dictionary is used to label the pixel. The final feature vector for each image is the frequency histogram of all texton labels in the dictionary.

To evaluate textures in an image, the image is similarly filtered with the filter bank. The filter response of each pixel is compared with entries in the texton dictionary, and the texton label of the closest match as calculated by Euclidean distance is used to label the pixel. The frequency histogram of the 300 textons is the final numerical descriptor of the image. This process is illustrated in Fig. 4 ▶.

#### 2.1.4. Validation of textons as a quantifier of droplet content   

**Figure 5:**
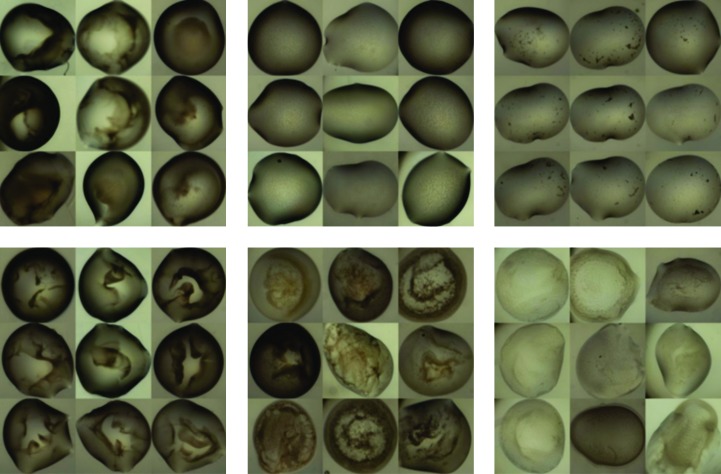
Examples of clusters of precipitation patterns. The clusters had visually consistent precipitation behaviour, indicating that the features produced can group similar precipitation patterns together.

The precipitation patterns of 1400 images randomly selected from 28 crystallization plates were clustered to examine whether the method is able to discriminate droplets based on their patterns. Hierarchical clustering with Ward’s minimum variance method (Gordon, 1987 ▶) was used, where clusters are merged to minimize the variance within clusters. The distance between droplets, *d*(*x*, *y*) was calculated using the χ^2^ distance between their histogram,
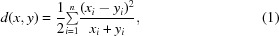
where *x_i_* and *y_i_* are the normalized bin counts of the *i*th feature of droplets *x* and *y*, respectively, *n* = 300 in this case and the distance is weighted inversely by the sum of the bin counts. The resulting 50 clusters were manually inspected and found to have visual consistency; examples of these clusters are shown in Fig. 5 ▶.

### 2.2. Image-processing pipeline   

**Figure 6:**
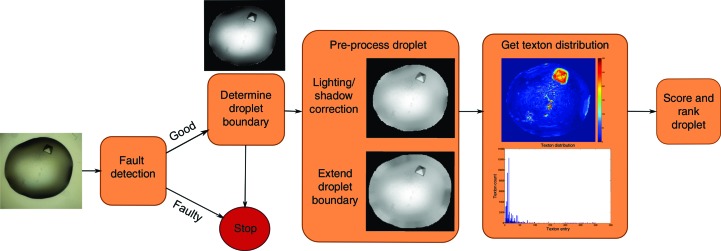
Overview of the image-processing pipeline. An image is first passed through a fault-detection system. If the droplet is not faulty, the droplet is segmented, corrected for lighting and shadow effects, and its boundaries are extended radially. Features are derived using the texton method (see §[Sec sec2.2]2.2) and are used to score the image with a random forest classifier.

Full analysis of droplets involves four main steps, as illustrated in Fig. 6 ▶, each of which is described in detail in the following sections: (i) fault detection, (ii) droplet segmentation, (iii) droplet pre-processing and (iv) calculation of texton distribution. Each new droplet image is first converted to greyscale and contrast-adjusted so that its grey levels cover the full spectrum of 0–255 and is passed through a fault-detection system, which identifies whether a droplet is acceptable or faulty (empty well, incomplete dispensing or camera faults). Faulty droplets are removed and are not further processed. For acceptable droplets, the droplet boundary is determined automatically and the segmented droplet is processed to correct for lighting and shadow effects around the boundary, and extra pixels are padded to extend the droplet radially. Finally, the texton distribution is calculated as described in the previous section. The pipeline was implemented in *MATLAB* with the *Image Processing Toolbox* and *Statistics Toolbox*.

#### 2.2.1. Well segmentation and fault detection   

**Figure 7:**
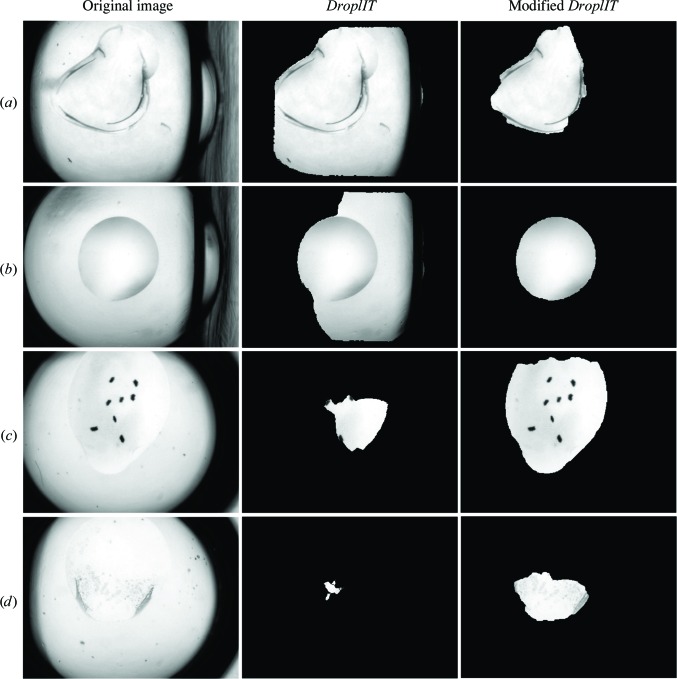
Comparison of droplet segmentation with *DroplIT* (Vallotton *et al.*, 2010 ▶; middle column) and the modified version (right column). *DroplIT* may under-segment the droplet, especially if the well frame has the characteristics in (*a*) and (*b*). (*c*) and (*d*) show more difficult examples where the droplet boundaries are almost invisible at certain parts. However, droplets are still segmented, and useful information can be found within the segmented region.

Errors owing to inaccuracies of the droplet-dispensing robots may result in empty subwells, unusually small droplets (where either the protein or reservoir solution was not added) or droplets sitting at the edge of the subwell. Errors in the imaging system may also produce images where the subwells are partially out of the field of view of the camera. To identify these failed droplets, background images were obtained for each of the subwells by taking the average of that subwell for all wells from an empty plate. This should be performed for every type of plate since it is plate-specific; in this case, we generated background images for the 3-Well Crystallization Microplate (SWISSCI), which is most commonly used at the SGC Oxford. This background image is rigidly registered to a new image by searching for the *x* and *y* translation that minimizes the pixel-to-pixel difference, giving the location of the well relative to the image. The area outside the well (the well frame) is masked and the remaining pixels are intensity-normalized to have a mean intensity of 0 and a standard deviation of 1. The following statistical descriptors are calculated from the gradient image of the normalized image: mean, standard deviation, skewness, kurtosis and the distribution of the absolute gradients at fixed 50 bin centres ranging from 0 to 50. These 54 features are used with the area, centroid, eccentricity (ratio of difference between foci and the major axis length), major and minor axis length of the segmented droplet (described next) as inputs to a random forest classifier which predicts whether a droplet image is good or faulty. The classifier used here was trained with 11 326 images (5225 faulty and 6101 non-faulty images) and has an accuracy of over 94%. The majority of inaccurate classification occurs in experiments set up with detergent leading to the much lower contrast of the droplet boundary in such droplets (Fig. 7 ▶ *d*).

#### 2.2.2. Droplet segmentation   

To segment the droplet, the well frame location as identified in the rigid registration step is used in a modified version of *DroplIT* (Vallotton *et al.*, 2010 ▶). *DroplIT* identifies closed contours around a point where the average pixel intensity along the contour of its gradient image is extremal. Images are transformed to the polar coordinates and the circular shortest path is computed. In contrast to the original method, which uses thresholds to identify the well frame, the well frame location is used instead to more reliably remove strong edges from the well frame and thus improve droplet segmentation. Comparisons of segmentation with the original and modified method are shown in Fig. 7 ▶.

#### 2.2.3. Pre-processing droplet: shadow correction   

**Figure 8:**
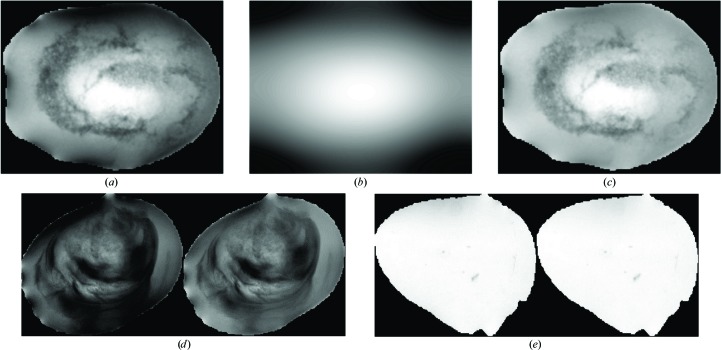
Gamma correction to correct for shadows around droplets. The droplet (*a*) is filtered with a low-pass filter, resulting in a blurred image as shown in (*b*). The normalized values of the low-pass filtered image are then used as γ values, resulting in the corrected image in (*c*). (*d*) and (*e*) show more examples of droplets before (left) and after (right) gamma correction. Shadow pixels along the edges of the droplet have been ‘boosted’, while the centre details remain relatively similar. Droplets with no dark edges should have no changes.

Shadows in drops cause artificial textures to be detected; they arise from lighting conditions and droplet morphology, and are sometimes enhanced by the presence of PEG in solution. To correct for shadows, a non-uniform gamma correction is applied over the droplet, which increases the intensity of darker pixels around the edge. Corrected pixel intensities 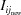 are calculated as
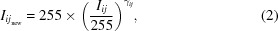
where 255 is the maximum intensity in a grey-scale image, *I_ij_* is the original pixel value at row *i* and column *j*, and
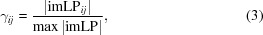
where imLP is the low-pass filtered image (with Gaussian low-pass filter, σ = 1) of the droplet. The γ value for each pixel is directly weighted by its original intensity. Lower γ values result in a higher intensity boost for darker pixels. Fig. 8 ▶ shows the process and outcome of gamma correction.

#### 2.2.4. Pre-processing droplet: droplet-boundary extension   

**Figure 9:**
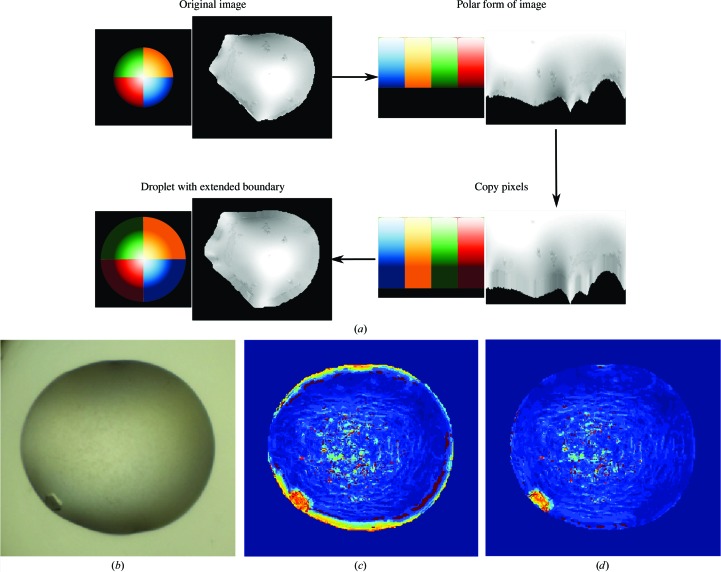
(*a*) Process of extending the boundary of a droplet. A colour wheel is shown beside the droplet for illustration. The segmented droplet is cropped and converted to its polar form. The droplet boundary is extended by replacing 13 pixels beyond the boundary in each column with the median of intensities in the ten pixels closest to the boundary in the corresponding column. The image is finally converted back to Cartesian space. The padded pixels should be similar to the boundary they were derived from, with some variations owing to interpolation errors. (*b*, *c*, *d*) Effects of droplet boundary. (*b*) A typical example of crystal growth at the edge of the droplet. (*c*) The strong edge of droplet boundaries gives a strong signal that often corresponds to crystal edges, either masking the presence of crystals by the edge or creating false-positive signals. (*d*) By extending the droplet boundary to avoid the strong edge, noise can be supressed for better crystal detection.

Filter responses at the edge of droplets will be dominated by the droplet boundary. To avoid the strong boundary effect, droplets are artificially extended radially and the filter response of this extended region is ignored in the final frequency count. Fig. 9 ▶ illustrates this operation. The cropped version of the segmented droplet is converted to its polar form centred at the droplet centroid. The polar image is of size *R* × 360, where *R* is chosen to be half of the largest dimension (*x* or *y*) of the original segmented droplet. For each column (1–360°) in the polar image the droplet boundary is extended by replacing 13 pixels (half of the filter size + 1) outside the radius of the droplet with the median of intensities of the ten pixels closest to the droplet boundary. The median is used instead of the mean to avoid outliers from possible imperfect segmentation. The new extended polar image is then converted back into rectangular space, resulting in a padded droplet. The ring of added pixels is copied to the original droplet image. This provides a sufficient number of neighbouring pixels for the calculation of filter responses for pixels in the original edge; the filter responses for pixels in the extended region are ignored in the final histogram count. Figs. 9 ▶(*b*), 9 ▶(*c*) and 9 ▶(*d*) show a comparison of texton labels with and without droplet extension.

#### 2.2.5. Calculation of texton distribution   

The gamma-corrected and extended image is intensity-normalized to have μ = 0 and δ = 1 for intensity invariance and is scaled by a factor of 0.25 to match the textons in the dictionary. The texton distribution is then calculated as described in §[Sec sec2.1.3]2.1.3. Computation time from reading an image to generating the texton distribution histogram is on average under 1.5 s on a Windows 7 machine with 8 GB of RAM and an Intel Core i5-2500.

### 2.3. Data sets and training algorithm   

#### 2.3.1. Data set   

At the SGC, crystallization experiment droplet images are automatically captured with a Minstrel HT system (Rigaku). Experimenters label images with scores between 1 and 10, where labels 1 and 2 are for precipitates, 3–5 for microcrystals and 6–10 for mountable crystals. It should be noted that labelling is optional, and it is usually only the interesting droplets that are labelled. Two sets of training images were selected: (i) ‘interesting’ images, a random subset of 2501 images of droplets that were given labels of 3 and above captured between April 2013 and July 2013, and (ii) ‘uninteresting’ images, a random subset of 3553 images from the same period with scores <3 or with no scores recorded.

#### 2.3.2. Training algorithm   

The set of 2501 ‘interesting’ droplets and 3553 ‘uninteresting’ droplets were used to train a two-class random forest classifier (Breiman, 2001 ▶) with 500 decision trees using the *TreeBagger* function of *MATLAB*. The random forest algorithm was chosen for its speed and its ability to deal with the large numbers of both instances and features, unlike competing algorithms. Given a set of features from new test images, the random forest produces the posterior probability for a particular class, which is used directly as scores for the images. In this case, for the ‘interesting’ class a score of 1 indicates that the droplet is likely to contain crystals or crystalline behaviour, while a score of 0 indicates that it is ‘uninteresting’. In a typical classification exercise, a threshold is set to determine which class the data point belongs to. Tenfold cross-validation of the classifier at a cutoff of 0.5 gave an average area under the ROC curve of 0.9418 ± 0.0027, indicating good separation of scores for both classes, and an average accuracy of 0.8930 ± 0.0044. However, as the intention is to rank and not to classify droplets, no threshold was selected; instead, the scores were used to rank droplets directly.

### 2.4. Validation and cross-imaging- platform application   

To test the algorithm, a separate set of images was selected from 196 plates set up at SGC Oxford over a different date range (July to September 2013) with at least one recorded crystal (label ≥3 as scored by SGC crystallographers). Of these, 101 plates were sparse-matrix screens, while the remaining 95 were optimization experiments. Each plate contained 96 wells with three subwells each, where the subwells share the same protein solution and reservoir solution at different mixing ratios (typically 2:1, 1:1 and 1:2). Droplets in these plates were ranked by the algorithm and the highest rank of crystals marked by crystallo­graphers was determined either by subwell or by well, where the maximum score of the three subwells was used.

**Figure 10:**
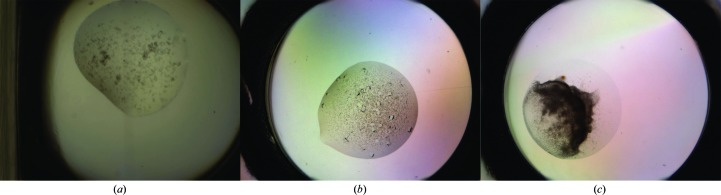
Comparison of images captured at (*a*) SGC, Oxford with the Minstrel HT and (*b*, *c*) Novartis, Basel with the Rock Imager. SGC droplets are captured at one focal depth, resulting in out-of-focus regions, whereas Novartis images are a combination of seven focal depths and hence are sharper across the droplet. The pllate material, seals and optics used at Novartis also produce colour gradients in the images.

For validation of the robustness of the algorithm across imaging platforms, we analysed images acquired by the Structural Biophysics group of the Novartis Institute for Biomedical Research (NIBR), Basel using a Rock Imager (Formulatrix). Images of 134 plates with at least one recorded crystal were selected. A comparison of this data set with that from the SGC is summarized in Table 1 ▶. These images were captured in the Extended Focus Imaging mode, where multiple focal depths are combined to form the final image, hence producing sharper images across the droplet. A combination of seal materials and polarizing optics also produces more colourful images. Furthermore, the resolution was just over half of that of the SGC at ∼2.99 µm per pixel length. Fig. 10 ▶ shows a comparison of SGC images and Novartis images. Background images for the type of plate used were obtained similarly as described in §[Sec sec2.2.1]2.2.1. To address the resolution difference, we scaled the images by a factor of 0.5 (instead of 0.25). Droplet segmentation proved to be more difficult owing to the sharp edges of precipitates within the droplets (see, for example, Fig. 10 ▶ *c*). When *DroplIT* failed on the original image, it was instead applied to a gamma-corrected image, as described in §[Sec sec2.2.3]2.2.3 (with a Gaussian low-pass filter, σ = 10), which blurs large, dark regions often associated with precipitates without affecting the high-frequency change of a droplet boundary, or to a blurred version of the image. This reduced the rate of failure of droplet segmentation from 12 to 2%. The same analysis was carried out, determining the highest rank of crystals marked by crystallographers.

### 2.5. Human evaluation of the crystallization outcome with the ranking system   

To show that such ranked viewing of images results in drops with microcrystals being more carefully evaluated, ten plates containing (micro)crystals were randomly selected from the SGC and divided into two sets (*A* and *B*). Each set consisted of three sparse-matrix screens and two optimization screens of different protein targets. Two groups of five crystallographers from the SGC with varying experience were asked to evaluate each plate in a standardized time interval of 2 min, which was insufficient to view all 288 droplets in a plate and thereby simulates the end goal of the program of minimizing the time required to identify all crystals. (Limiting the time commitment to 20 min also helped in recruiting sufficient volunteers.) The first group viewed set *A* in a ranked order and set *B* in an unranked order, while the second group viewed set *B* in a ranked order and set *A* in an unranked order. The majority vote (microcrystals or crystals) of each group of crystallo­graphers was used to compare against the other group for missed (micro)crystal annotations.

## 3. Results and discussion   

### 3.1. Ranking *versus* classification or filtering   

Although previous studies set out to classify crystallization drops into discrete human-assigned categories, it is not clear that they demonstrated that this is achievable or indeed that the underlying premise of one-drop-one-score is even useful: individual drops routinely exhibit multiple precipitation behaviours that may inform one another but which are nevertheless only very loosely defined by the community (Newman *et al.*, 2012 ▶). Unsurprisingly, even human classification of droplets yields poor agreement rates (Buchala & Wilson, 2008 ▶), and using such variable opinions as ground truths severely undermines the training of learning algorithms and not only reduces accuracy, especially for multi-class classifiers, but makes it unmeasurable. The increased rate of false negatives is particularly pernicious since the formation of crystals is in general a rare event yet is experimentally crucial to detect.

Here, we target a more realistic goal, namely to prioritize droplets for viewing: we judge this to be more useful because it does not pre-empt decisions but helps them to be made more accurately as well as more rapidly. One version of this approach is filtering, as employed, for example, in Rigaku Automation’s viewing software for images captured on a UV-enabled instrument, or more recently by Mele *et al.* (2014 ▶), who filter out images from further examination based on the lack of change (differences) in a droplet over time, on the basis that such changes may indicate the formation, growth or disappearance of crystals. However, filtering is merely an extended case of classification, where instead of a single classification cutoff a tuneable cutoff or criterion is still required to hide a subset of data.

In contrast, ranking circumvents the problem of selecting filtering cutoffs or criteria, which tend to be arbitrary; instead, it rearranges the data in a more meaningful way. In our approach, we rank droplets on a continuous scale (0 to 1) of their probability of being ‘interesting’: this means that crystals or microcrystals are likely to be viewed first, while the ‘uninteresting’ droplets (precipitates, clear drops) are viewed later. In the real-world environment where time is limited and attention level decreases with time, the ranking system focuses resources on what is most likely to matter, namely the likely presence of crystallinity.

The ranking score is obtained by repurposing a two-class classifier trained on images assigned manually into only two categories, namely containing crystallinity (‘interesting’) or not (‘not interesting’), and therefore containing the respective textons. When applied to new images, the classifier generates a score (0 to 1), which ordinarily would be compared against a threshold in order to assign images to either category; instead, here the score is directly employed for sorting a given set of images (typically all images in a crystallization plate).

Multi-class classifiers can in principle also be used to rank images, most trivially by assigning each class to a rank (*e.g.* ‘crystal’ over ‘microcrystal’ over ‘spherulite’ and so on); however, this ranking is algorithmically arbitrary (for example, should ‘clear’ drops be viewed before ‘precipitate’ drops?), and moreover it is non-obvious how to rank images within classes, since the classifier scores are no longer uni­directional. On the other hand, Buchala & Wilson (2008 ▶) have shown that reducing the number of classes increases the human agreement rate and hence the accuracy of the classifier, suggesting that the fewest possible classes (two) would support the highest reliability.

### 3.2. Algorithm performance   

**Figure 11:**
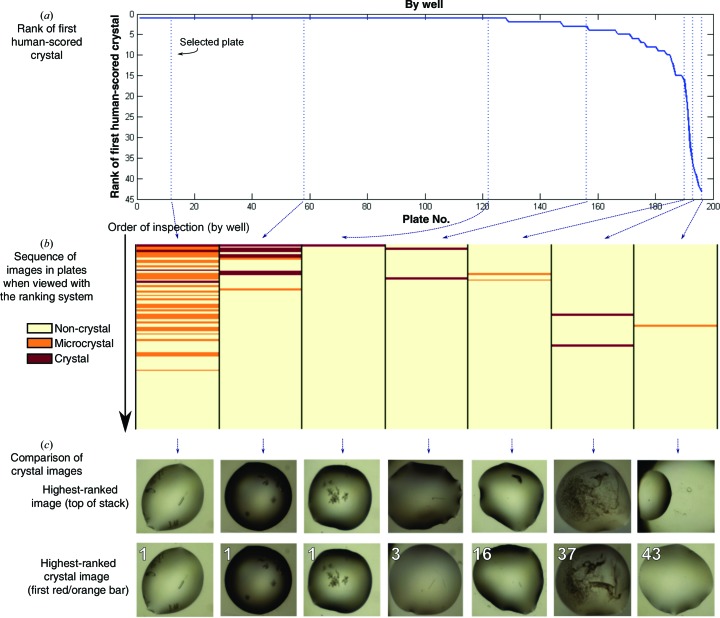
Effectiveness of the algorithm at ranking crystal-containing images. (*a*) Rank of first human-scored crystal image in the plate. The ideal curve is a horizontal line with *y* axis = 1 if all plates have been well ranked. (*b*) Enrichment plot for the representative plates marked by the dotted lines in (*a*). Each column represents a plate with the wells as horizontal lines coloured for clear crystals (red), microcrystals (orange) and ‘uninteresting’ droplets (yellow), as scored by a crystallographer; it is clear that ‘interesting’ images are effectively being moved to the top of the viewing order. A similar plot for all plates used can be found in Supplementary Fig. S3. (*c*) Actual images for the plates shown in (*b*), showing the inherent problems in those drops which the algorithm failed to rank highly (the last three).

Our approach appears to rank images effectively, if judged by criteria that are reasonable in routine laboratory usage: for plates where wells were viewed with our new ranking order, the first well was a crystal-containing image for 128 out of a total of 196 plates (65.31%). The number of such ‘successful’ plates increases if the criterion is relaxed to expect at least one crystal in the top 10 or top 32 ranked wells (Table 2 ▶), as also illustrated by the curve in Fig. 11 ▶(*a*), which shows that for most plates the first human-annotated crystal is very high in the ranking order. We show both the results for viewing by well and subwell in Table 2 ▶: while viewing by subwell (single droplets) is the common practice, when all drops in a well have the same chemical composition and differ only in the mixing ratio (§[Sec sec2.4]2.4) there is added value in viewing all of the subwells of the well side by side, since precipitation trends can be directly observed.

**Figure 12:**
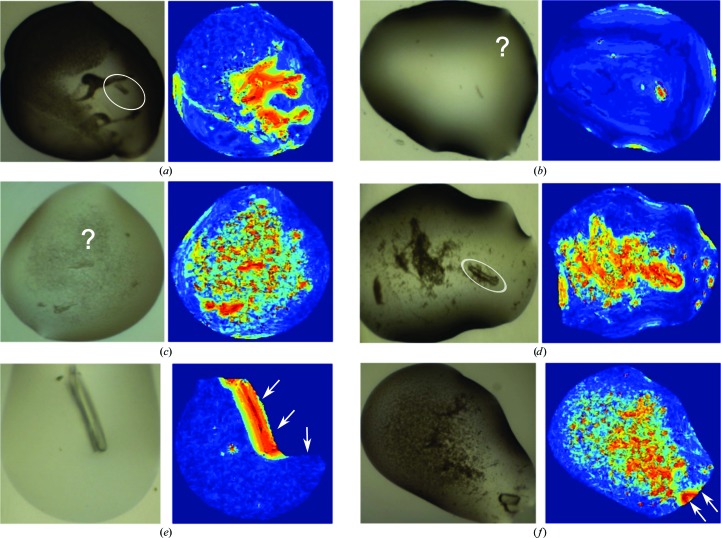
Examples of images and the corresponding texton maps of annotated crystals and microcrystals that received low ranks. Such crystal images were usually out of focus (*a*), questionably or generously labelled (*b*, *c*) or surrounded by precipitates (*d*), or the droplet was inaccurately segmented (*e*, *f*).

It is not only the top drop but all drops in a plate that seem to be effectively ranked: Fig. 11 ▶(*b*) and Supplementary Fig. S3 illustrate how the top of the viewing order is generally enriched in images with crystals and microcrystals. Moreover, where the ranking failed to move the human-annotated crystal to the top, it was usually questionable whether the images had been correctly marked (Fig. 11 ▶ *c*) or else the images themselves were problematic (Fig. 12 ▶). As with all learning approaches, the performance is expected to improve as more images are added to the training set; but we conclude that the method does push at least one crystal to the top with high likelihood.

In our calculation of texton distribution, the closest match of the filter response of a pixel to the dictionary entries was calculated with the Euclidean distance, which is the commonly used distance measure in texton research and hence is a natural choice for this initial work. At the same time, distance measures remain an unresolved question in the field, and there is no *a priori* reason to believe the Euclidean metric is meaningful for this application; thus, exploring other distance measures, and identifying those that are consistent with human perception, may significantly improve the performance of the algorithm; this will be addressed in future work.

### 3.3. Performance for different imaging systems   

**Figure 13:**
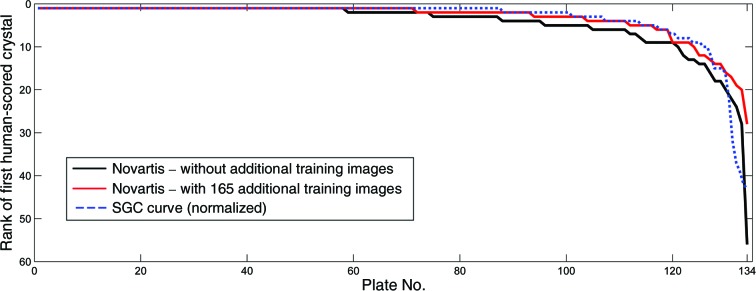
Rank of the first human-scored crystal image in the plate for the Novartis data set of 134 plates. The black curve shows the performance of direct application of the ranking system without additional training images. By adding 165 (15 crystal and 150 noncrystal) images acquired with the Rock Imager, more crystal images were ranked higher, as shown by the red curve. The SGC performance curve (blue) is normalized to an equal length for qualitative comparison.

The texton approach is robust enough to work well on different imaging systems without additional training. Using the learning parameters obtained with images acquired by the Minstrel HT system at SGC Oxford, the method was transferred to Novartis, Basel, which has a Rock Imager system. The transfer was generally straightforward, apart from the modifications in droplet segmentation as mentioned previously and a different scale factor (0.5 instead of 0.25 at the SGC) to approximately match the resolution at the filtering stage. Table 3 ▶ and Fig. 13 ▶ show the performance of direct application of the system, as well as the same system with additional training images (15 ‘interesting’ and 150 ‘uninteresting’ randomly selected images), compared with the SGC Oxford data sets; as expected, the results improved with more training images. Note that a direct comparison of the SGC and Novartis numbers is not meaningful for this number of plates; in addition the ratios of sparse-matrix screens and optimization screens are different.

This result indicates that the texton approach does not need a very high level of detail in the images: SGC and Novartis images have similar field of view (the entire subwell of a typical SBS-format crystallization plate; Fig. 10 ▶) but different pixel resolutions, yet the final scaling is at lower resolution than both for the texton analysis. The more important factor presumably is that the depth of field encompasses the majority of the precipitation behaviour, although no data set is yet available to test this.

### 3.4. Scores as a profile of the plate   

**Figure 14:**
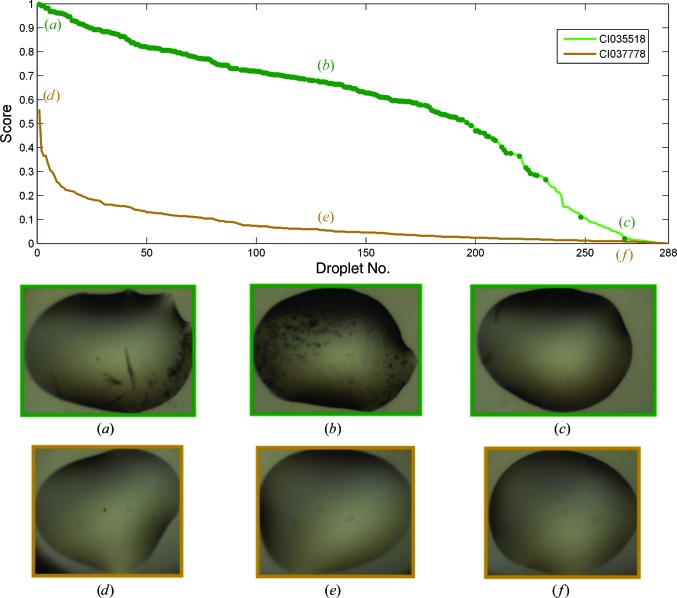
Comparison of scores of droplets in two plates. The scores are the posterior probability of a droplet being ‘interesting’ and the profile of the curve gives an overview of a plate. Plate CI035518 (green) was an optimization screen, in which 213 droplets contained crystals or microcrystals, marked with dark green dots on the curve. It thus received generally higher scores. In contrast, CI037778 (brown) contained no crystals and contained mainly clear drops. The scores for this plate tailed off rapidly. The highest scored image here was a droplet with dust speckles. The embedded images are the corresponding droplets that that were ranked 1, 100 and 268.

The collective scores of droplets across a plate form a profile of the plate and can be used to make quick judgements. Since each droplet score is the posterior probability of the droplet being ‘interesting’, a plate with many wells containing crystals will have a different profile from one with none. Fig. 14 ▶ shows the different profiles of a plate with 244 crystals and one with no crystals. The profile can also be used to determine a cutoff for images to inspect: across the 196 plates analysed, over 88% of the plates would have at least one crystal found by just inspecting images with scores greater than 0.5. This also corresponds to not inspecting an average of 90% of ‘uninteresting’ droplets in the plate. Table 4 ▶ shows the trade-off between the percentage of plates with at least one crystal found and the number of uninteresting droplets to view, which serves as a confidence indicator for the various cutoff values.

### 3.5. Effectiveness of drop ranking for human scoring   

An aspect that has not been much explored in the existing literature is how to present most effectively the computed classifications, or for that matter the features obtained from alternative imaging techniques (SONICC or UV). A common method in vendor software is to label the images with tags and different colour schemes (Mele *et al.*, 2014 ▶), and sophistication is introduced to allow users to sort by labels or to hide subsets of data. Nevertheless, images remain arranged based on their physical location on the crystallization plate, even though intuitively this does not seem to be the most efficient way of viewing images.

**Figure 15:**
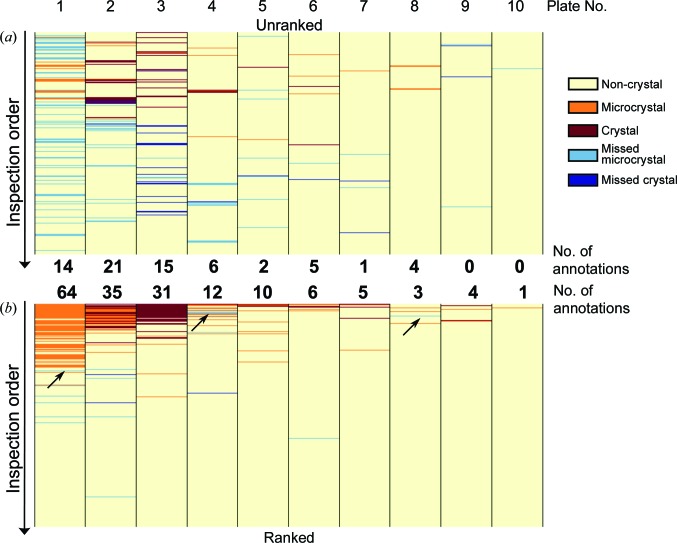
Comparison of annotations between two groups of crystallographers. Each column represents a plate of 288 droplets inspected in the unranked order (*a*) and ranked order (*b*), and the rows are coloured according to the majority vote for these images: yellow, non-crystals; orange, microcrystals; red, mountable crystals. Blue rows indicate missed (micro)crystals, where the corresponding images were annotated in one viewing order but not the other. The total numbers of annotations (orange and red rows) are shown in between. The black arrows show the location of missed (micro)crystals in the ranked order.

We show here that enriching the number of crystal-containing images to inspect first does indeed result in closer attention apparently being given to interesting droplets. We tested this by comparing how effectively crystallographers could evaluate crystallization plates under extreme time pressure (2 min per plate, ten plates) both with or without the ranking system. All plates were viewed by two groups of five crystallographers, with plates viewed in rank order by one group being viewed unranked by the other, but with both groups performing both ranked and unranked viewing; the consensus score within each group was taken to reduce human error (Fig. 15 ▶; individual scores are given in Supplementary Fig. S4). As expected, more (micro)crystals were annotated when the images were viewed in ranked order, and where crystallinity was rare (plates 9 and 10) unranked viewing resulted in (micro)crystals being missed, even those located early in the early viewing order, in contrast to the ranked viewing: evidently images are considered more carefully if they are known to be likely to be interesting. At the same time, ranked viewing does not appear to introduce more generous scoring at the top of the rank through confirmation bias, because microcrystals were also missed in ranked viewing (Fig. 15 ▶ *b*; blue rows in plates 1, 4, and 8, black arrows); on the contrary, annotation appears to become more stringent if larger (micro)crystals have already been observed.

### 3.6. Implementation, deployment and availability   

**Figure 16:**
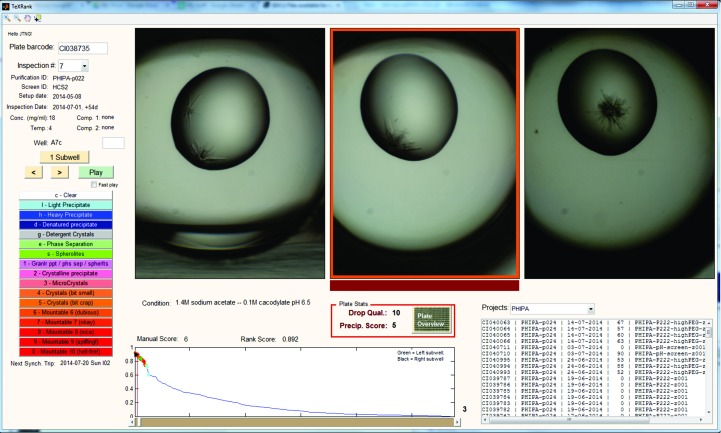
Snapshot of *TeXRank*, the custom viewer written to display images of a given plate in the ranked order. The plot at the bottom shows the scores of droplets, giving a quick overview of the plate. We also display three subwells of a well together, where each subwell contains the same precipitant and protein sample at different mixing ratios. The image to be scored is highlighted with a red bar at the bottom, and the scoring system and experiment information mirror the software that users are accustomed to.

Since rearranging image order is fundamental to our approach, and given that the profile of scores is so informative, we developed a custom viewer (Fig. 16 ▶), because vendor-supplied viewers do not in general allow the necessary customizations. The viewer has been integrated with the existing database and data-storage infrastructure so that users can view the information of a plate and annotate droplets as they would normally with vendor software. Furthermore, the viewer has the option of viewing subwells of a well side by side: this is very informative where the subwells are the same experiment (protein plus reservoir solution) at different mixing ratios (the default method for coarse screens at SGC Oxford): the juxtaposition allows the immediate evaluation of precipitation trends across the different protein concentrations. Various features of known usefulness have also been added to ensure that the software is fully functional for routine use.

The algorithm and custom viewer have been deployed at both SGC Oxford and the Structural Biophysics group, Novartis, Basel on 64-bit Windows 7 machines. The algorithm runs on an hourly basis, analysing all plates inspected in the previous hour. At the SGC, the average processing time per plate of 288 images is under 6 min on an Intel Core i5-2500 CPU with 8 GB RAM, which is well below the image-acquisition time of ∼10 min per plate with the Minstrel HT, and causes no backlog in processing even though two imagers are running concurrently. At Novartis, a plate of 96 images is processed in less than 4 min on an Intel Xeon X5550 CPU with 8 GB RAM, which also easily accommodates the typical image-acquisition time of less than 6 min for 96 wells with the Rock Imager.

The software is available as a standalone package, *TeXRank*, requiring only the freely available *MATLAB* Compiler Runtime (MCR); however, full integration with existing database and imager systems and deployment of the viewer will invariably require bespoke effort. The executable for image processing and generating ranking scores requires little effort to run once the reference background images have been generated, but it is the viewer that must be set up to fit into the infrastructure of a given laboratory; an application programming interface (API) is under development to simplify this process. We have shown that integration is possible for both the Rigaku and the Formulatrix systems, and for these particular versions of the systems it could take as little as 3 days to integrate fully for someone familiar with either system and with general database knowledge. The code and executables can be downloaded from https://github.com/thesgc/TeXRank.

## 4. Conclusions   

We show that textons are effective at describing crystallization experiments objectively with fast computation times, making them suitable for everyday use. These descriptors are effective for ranking droplets by their probability of containing crystals or microcrystals, and the algorithm was transferable between two widely used commercial imaging systems for sitting-drop vapour-diffusion experiments. The collection of scores across a plate forms a useful profile for a quick overview of the suitability of the protein for structural studies; the algorithm and the custom viewer can be integrated into the existing infrastructure and are freely available. We have also shown that with the system, crystallographers have been able to identify crystals and microcrystals more efficiently and accurately by prioritizing which images to view and spend more time on.

## Supplementary Material

Supporting Information.. DOI: 10.1107/S1399004714017581/nj5198sup1.pdf

## Figures and Tables

**Table 1:** Comparison of validation data sets acquired from SGC, Oxford and Novartis, Basel

	SGC	Novartis
Total No. of plates	196	134
Sparse-matrix screens	101	114
Optimization screens	95	19
Scoring criteria		
Interesting	Label ≥ 3	Label = {Phase Separation, Salt, Microcrystals, Needles (one-dimensional), Plates (two-dimensional), Crystals (three-dimensional), Interesting}
Uninteresting	Label < 3	Label = {Clear, Precipitate}
Additional differences		
No. of subwells used	3	1
Imaging mode	Single focus depth	Extended focus imaging
Optics	No polarization effect	Visible polarization effect
Resolution (µm per pixel length)	∼1.13	∼2.99

**Table 2:** A comparison of the performance of the algorithm before and after review of image annotations The top and bottom halves of the table show the number and percentage of plates where the first marked crystal was ranked in the plate, in terms of ranking by well and ranking by droplet, respectively.

	No. of plates	%
Rank (well)		
1	128	65.31
<10	185	94.39
<32	192	97.96
Rank (droplet)		
1	118	60.20
<29 (10%)	189	96.43
<72 (25%)	191	97.45
Total No. of plates	196	

**Table 3:** Percentage of plates according to the rank of the first human-scored crystal for the SGC and Novartis data sets

Rank	% of plates (SGC)	% of plates (Novartis, without additional training images)	% of plates (Novartis, with 165 additional training images)
1	65.31	43.28	52.99
<10	94.39	90.30	92.54
<32	97.96	99.25	100.00

**Table 4:** Percentages of plates with at least one crystal found for different cutoffs of scores for viewing and the corresponding average of uninteresting droplets that were not inspected

Cutoff	Plates with ≥1 crystal found (%)	Mean % of uninteresting droplets unseen in a plate
0.8	60	98 ± 7
0.5	89	90 ± 12
0.2	99	59 ± 24
0.1	100	36 ± 23
0.05	100	20 ± 17
0.01	100	4 ± 6

## Footnotes

1 Supporting information has been deposited in the IUCr electronic archive (Reference: NJ5198).
