# Supplementary material for: Using textons to rank crystallization droplets by the likely presence of crystals
Source: Acta Crystallogr D Biol Crystallogr. 2014 Sep 27;70(Pt 10):2702–18. doi: 10.1107/S1399004714017581 (PMC4188010; doi:10.1107/S1399004714017581)
Supplement: Supplementary file 1 [file d-70-02702-sup1.pdf]

Acta Cryst D

BIOLOGICAL CRYSTALLOGRAPHY

Volume 70 (2014)

Supporting information for article:

Using textons to rank crystallization droplets by the likely presence of crystals

Jia Tsing Ng, Carien Dekker, Markus Kroemer, Michael Osborne and Frank von Delft

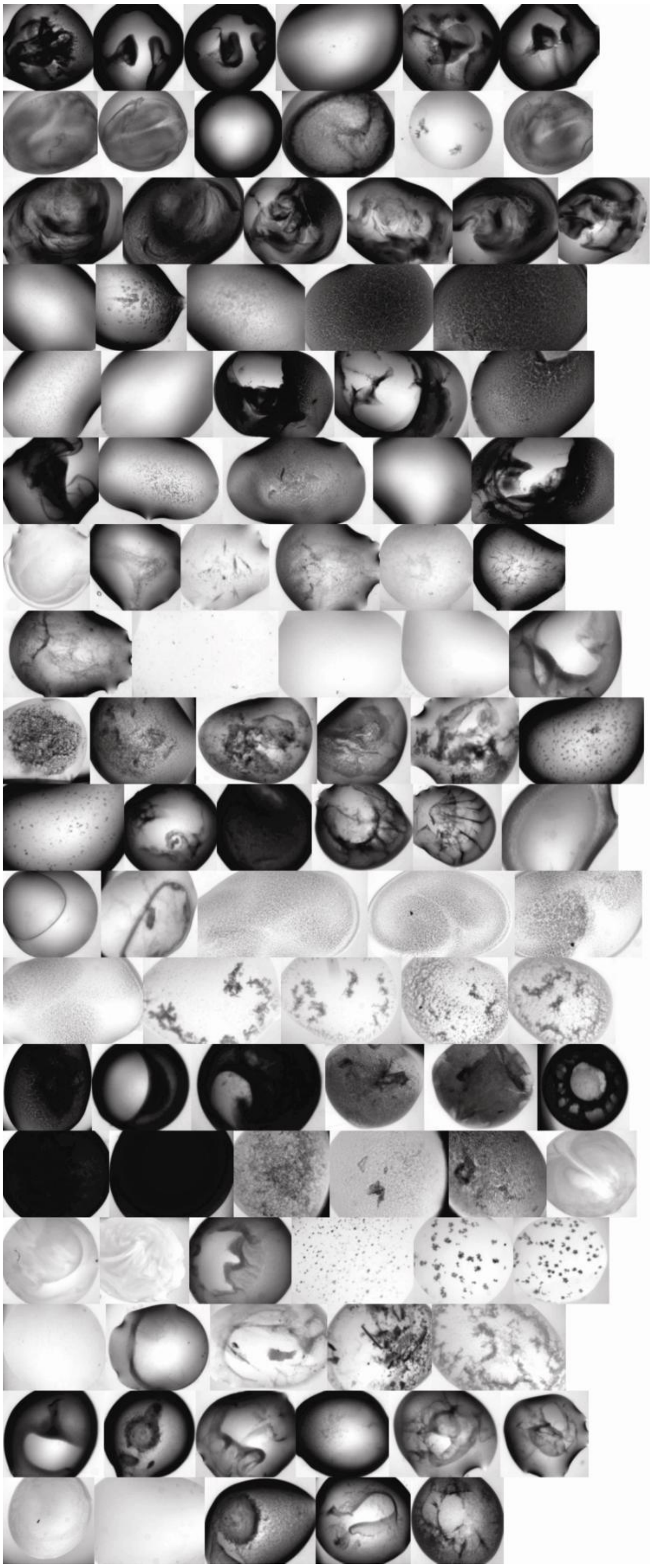

Figure S1 The 100 precipitation images used to develop the texton dictionary. 239 texton entries were derived by clustering the filter responses of these images.

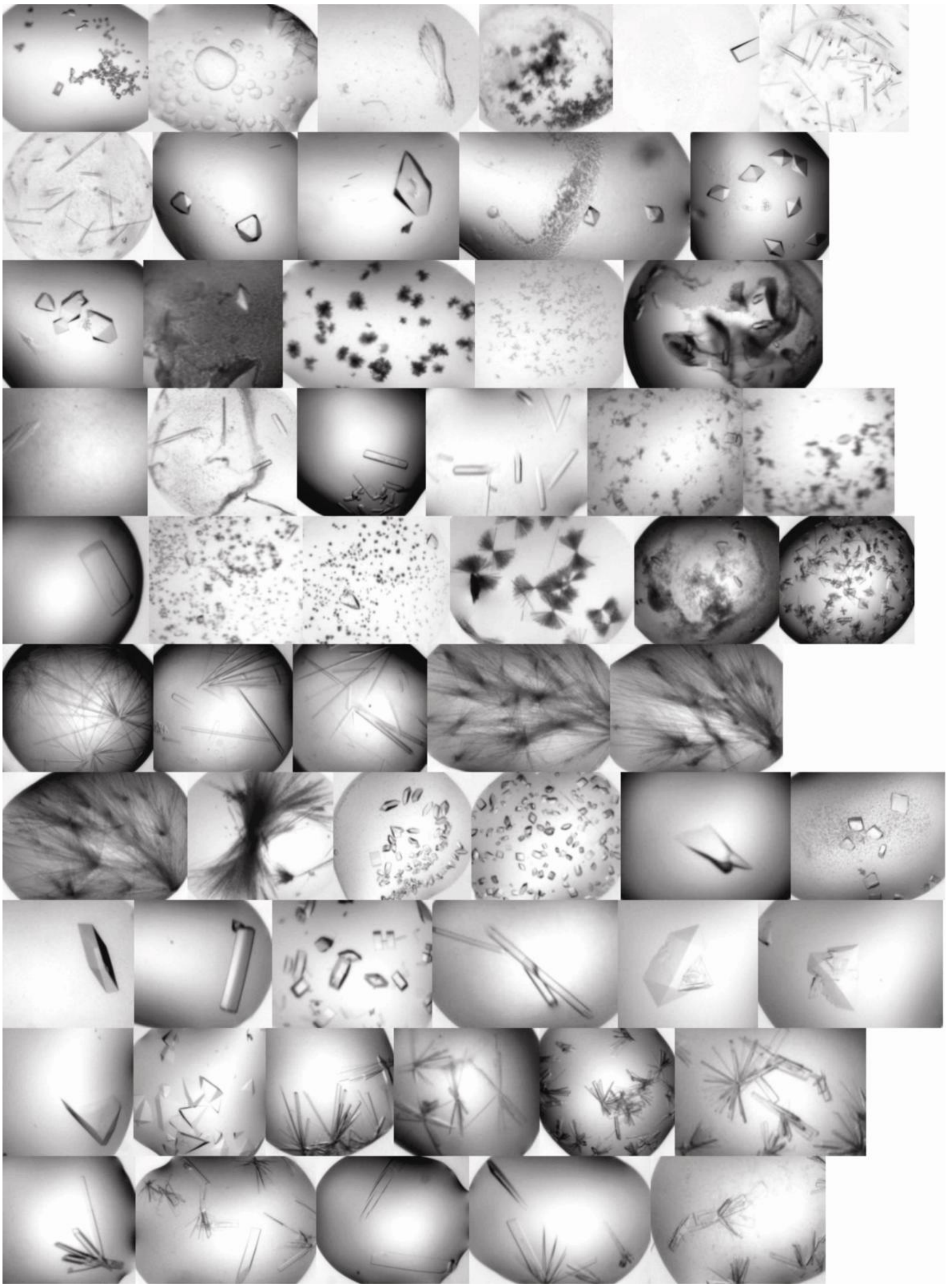

Figure S2 The 52 crystal-containing images used to develop the texton dictionary. 61 textons were derived from the filter responses of these images.

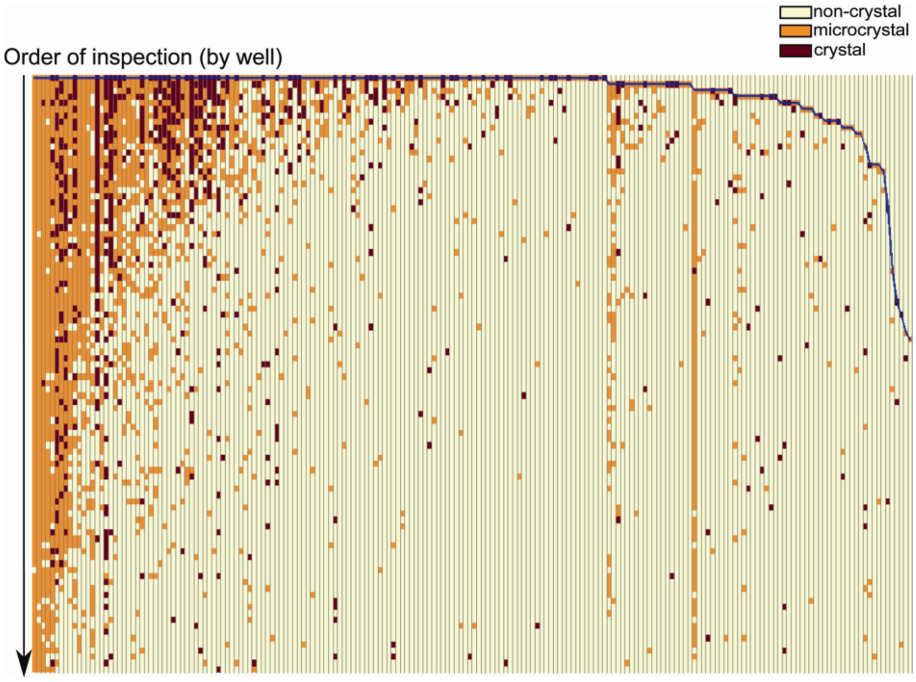

Figure S3 Position of all human-scored crystal images in the test set of 196 plates. Each column represents a plate and each row represents a well to be viewed. The blue curve marks the highest rank of human-scored crystal image for each plate, and is similar to Figure 11(a).

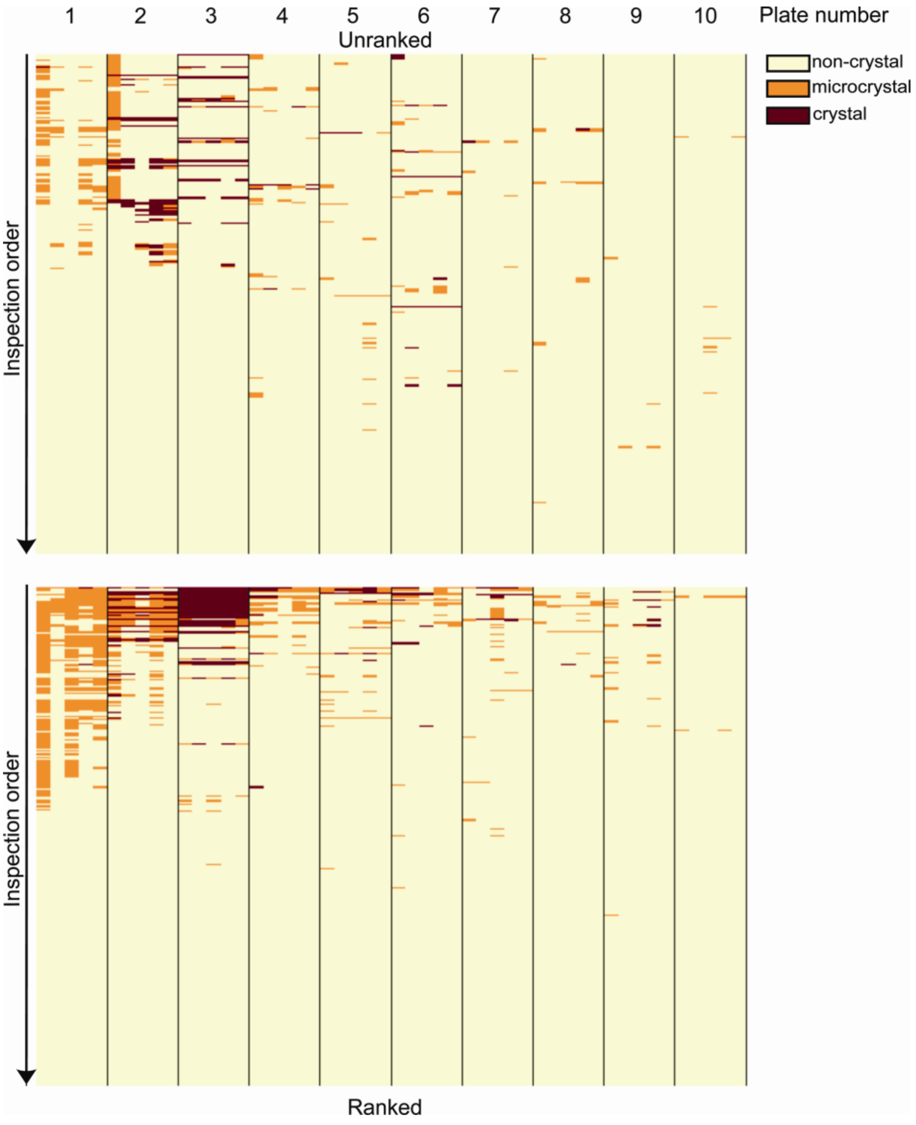

Figure S4 Individual annotations by the ten crystallographers, as represented by the coloured sub-columns. The top figure shows the annotations of the group of 5 crystallographers when viewing the plates in an unranked order, while the bottom figure shows the annotations of the crystallographers when they viewed the plates in the ranked order. The majority vote is used for Figure 15 and if there was a tie, the following priority was used: crystal, microcrystal, non-crystal.
